# Supplementary material for: Splice-Junction-Based Mapping of Alternative Isoforms in the Human Proteome
Source: Cell Rep. Author manuscript; Available in PMC 2020 Jan 15. (PMC6961840; doi:10.1016/j.celrep.2019.11.026)
Supplement: 2 [file NIHMS1546469-supplement-2.zip › DF1/PXD006675/VentricularSeptum/VentricularSeptum_30_PRPF40B_TLLQQLDDHGPMEPGIPPPFPPMGL.pdf]

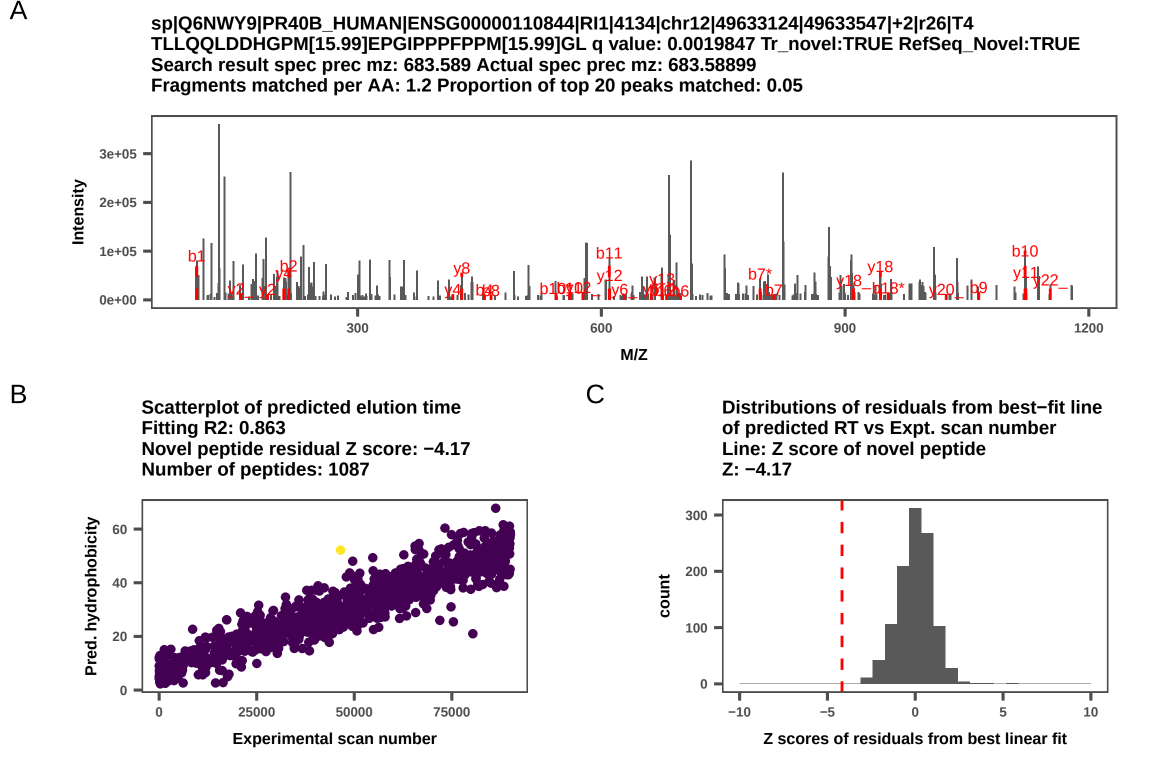
A
sp|Q6NWY9|PR40B_HUMAN|ENSG00000110844|RI1|4134|chr12|49633124|49633547|+2|r26|T4
TLLQQLDDHGPM[15.99]EPGIPPPFPPM[15.99]GL q value: 0.0019847 Tr_novel:TRUE RefSeq_Novel:TRUE
Search result spec prec mz: 683.589 Actual spec prec mz: 683.58899
Fragments matched per AA: 1.2 Proportion of top 20 peaks matched: 0.05
Intensity
3e+05
2e+05
1e+05
0e+00
b1
b2
y8
b11
y12
b7*
y18
b10
y11
y22
b7
b9
y20
M/Z
300
600
900
1200
B
Scatterplot of predicted elution time
Fitting R2: 0.863
Novel peptide residual Z score: −4.17
Number of peptides: 1087
Pred. hydrophobicity
60
40
20
0
0
25000
50000
75000
Experimental scan number
C
Distributions of residuals from best−fit line
of predicted RT vs Expt. scan number
Line: Z score of novel peptide
Z: −4.17
count
300
200
100
0
−10
−5
0
5
10
Z scores of residuals from best linear fit
